# Dissipation, Residues and Dietary Risk Assessment of Fluxapyroxad and Trifloxystrobin in Chilli Peppers

**DOI:** 10.3390/foods15213818

**Published:** 2026-10-26

**Authors:** Pengcheng Ren, Hongwei Zhang, Jing Jin, Chunyong Li, Junli Cao, Yanli Qi, Jindong Li, Shu Qin

**Affiliations:** 1 Shanxi Key Laboratory of Evaluation of Agro-Product Quality and Safety, Shanxi Center for Testing of Functional Agro-Products, Shanxi Agricultural University, Taiyuan 030031, China; 2 College of Plant Protection, Shanxi Agricultural University, Taiyuan 030031, China

**Keywords:** fluxapyroxad, trifloxystrobin, metabolites, chilli pepper, dissipation, terminal residue, dietary risk assessment

## Abstract

Investigating residues of fluxapyroxad and trifloxystrobin in chilli peppers is essential for food safety. In accordance with Good Agricultural Practice (GAP), supervised field trials were conducted to systematically evaluate the residue behavior and dietary exposure risks of these fungicides in chilli peppers. A validated method combining QuEChERS sample preparation with ultra-high-performance liquid chromatography–tandem mass spectrometry (UPLC–MS/MS) was developed to simultaneously quantify fluxapyroxad, trifloxystrobin, and their metabolites in chilli peppers. The method provided satisfactory recoveries of 92–101% with relative standard deviations (RSDs) below 6%, and the limit of quantification (LOQ) was 0.01 mg/kg for both analytes. Field-trial results showed both fungicides dissipated rapidly in peppers, following first-order kinetics with half-lives of 2.4–5.2 days for fluxapyroxad and 1.8–4.6 days for trifloxystrobin. The terminal residues of fluxapyroxad and trifloxystrobin ranged from <0.010 to 0.23 mg/kg and from 0.014 to 0.49 mg/kg, respectively. The chronic risk quotients of fluxapyroxad and trifloxystrobin were 92.57–92.61% and 13.27–13.30% of their respective acceptable daily intake (ADI), indicating that the dietary risks were acceptable. This study provides residue data and scientific evidence to support establishing and revising maximum residue limits (MRLs) for fluxapyroxad and trifloxystrobin in chilli peppers, as well as the safe use of both fungicides in China.

## 1. Introduction

Chilli pepper (*Capsicum annuum* L.) has significant economic value and is widely cultivated worldwide because of its rich nutritional profile, distinct flavour and versatile culinary applications [1]. Chilli peppers contain diverse bioactive compounds, such as vitamin C, carotenoids, phenolics, and capsaicinoids, that confer substantial health benefits through their antioxidant, anti-inflammatory and metabolic-regulatory properties [2]. China is the world’s largest producer and consumer of chilli peppers. According to the Food and Agriculture Organization (FAO), the harvested area of green chillies and peppers in China reached 770,656 ha in 2024, with a total production of approximately 17.31 million tonnes (https://www.fao.org/faostat/en/#compare, accessed on 6 July 2026). However, intensive cropping systems, combined with warm, humid growing conditions, make chilli pepper plants highly susceptible to a range of fungal pathogens, most notably anthracnose [3,4], powdery mildew [5,6], and gray mold [7,8], which can cause severe yield losses and deterioration of fruit quality if left unmanaged.

Within integrated disease management strategies, fungicides remain an important control measure because of their high efficacy, operational reliability and cost-effectiveness. Fluxapyroxad and trifloxystrobin have attracted considerable attention because of their complementary modes of action and broad-spectrum antifungal activity [9]. Fluxapyroxad, a pyrazole-4-carboxamide fungicide developed by BASF, belongs to the succinate dehydrogenase inhibitor (SDHI) class (FRAC group 7). It competitively inhibits succinate dehydrogenase (complex II) in the fungal mitochondrial respiratory chain, thereby disrupting electron transport and ATP synthesis and suppressing spore germination, germ-tube elongation, and mycelial growth [10,11]. Trifloxystrobin, an oximinoacetate strobilurin of the quinone-outside-inhibitor (QoI) class (FRAC group 11), binds to the Qo site of the cytochrome bc_1_ complex, blocking electron transfer and ATP production and thereby disrupting fungal energy metabolism [12,13]. Combining these two active ingredients provides a dual-mechanism approach that can enhance disease-control efficacy and, when embedded in an appropriate resistance-management program, delay the development of fungicide resistance.

Despite their agricultural benefits, the extensive application of these fungicides has raised persistent concerns about pesticide residues in food products and their associated risks to human health [14]. While several studies have investigated the dissipation and residue behavior of trifloxystrobin or fluxapyroxad individually in various crops, there has been limited focus on the simultaneous monitoring of both fungicides and their metabolites, especially in multi-site field validations, along with comprehensive dietary risk assessment. Previous research has provided valuable insights into the fate of these compounds within specific crops. Cui et al. [15] examined the dissipation of fluxapyroxad, mandipropamid, and mefentrifluconazole in chilli peppers, highlighting the effects of washing and drying. Ahlawat et al. [16] conducted field trials on cucumber at a single site in India, reporting half-lives for fluxapyroxad and trifloxystrobin. Similarly, Cui et al. [17] investigated fluopyram and trifloxystrobin in both cucumber and cowpea, alongside a preliminary dietary risk assessment. In green chilli, Kousik Mandal et al. [18] noted that fluopyram was not detected, and trifloxystrobin acid was detected only on days 0 and 3, suggesting that the recovery of metabolites and the coverage over time may require further optimization. Neeraj Sharma et al. [19] reported residue levels in onions; however, their analysis was confined to a single crop and lacked multi-location field data as well as a systematic health risk evaluation. Critically, to the best of our knowledge, no published method currently enables the simultaneous determination of fluxapyroxad, its two major metabolites, trifloxystrobin, and its acid metabolite across multiple agroecological regions. Furthermore, a comprehensive dietary risk assessment based on terminal residue data generated under diverse field conditions has yet to be conducted.

To address these gaps, this study aimed to (i) develop a sensitive and reliable ultra-high-performance liquid chromatography–tandem mass spectrometry (UPLC–MS/MS) method, coupled with a modified QuEChERS extraction procedure, for the simultaneous determination of fluxapyroxad, trifloxystrobin, and their metabolites (M700F008, M700F048, and CGA321113) in chilli peppers; (ii) evaluate the terminal residues of fluxapyroxad, trifloxystrobin, and their metabolites in chilli peppers at harvest under field conditions; and (iii) assess the dietary risks for the general population in China.

## 2. Materials and Methods

### 2.1. Chemicals and Reagents

Reference standards of fluxapyroxad (purity, 99.90%), trifloxystrobin (purity, 99.30%), M700F008 (purity, 99.5%), M700F048 (purity, 96.7%), and CGA 321113 (trifloxystrobin acid; purity, 98.68%) were obtained from Dr. Ehrenstorfer GmbH (Augsburg, Germany) and BASF SE (Ludwigshafen, Germany). HPLC-grade acetonitrile, methanol, formic acid, and ammonium acetate were purchased from Thermo Fisher Scientific (Waltham, MA, USA). Analytical-grade anhydrous magnesium sulfate and sodium chloride were obtained from Tianjin Damao Chemical Reagent Factory (Tianjin, China). Primary secondary amine (PSA, 40–60 μm) and graphitized carbon black (GCB, 120–400 mesh) were supplied by Agela Technologies (Tianjin, China). Purified water was supplied by Watsons Group (Guangzhou, China).

Individual standard stock solutions, each with a concentration of approximately 1000 mg/L, were prepared by dissolving 10.0 mg of each reference standard in acetonitrile to a final volume of 10 mL in volumetric flasks. The stock solutions were subsequently stored at ≤−18 °C. Matrix-matched calibration standards were created by performing serial dilutions of the combined intermediate standard solutions with blank pepper matrix extract and 5% formic acid in acetonitrile (*v*/*v*).

### 2.2. Field Experiments and Sampling

Supervised field trials were conducted during the 2024 and 2025 growing seasons at 12 representative chilli pepper production sites across China. The locations included Shenyang (Liaoning Province), Taiyuan (Shanxi Province), Weinan (Shaanxi Province), Beijing (Beijing Municipality), Xinxiang (Henan Province), Qingdao (Shandong Province), Songjiang (Shanghai Municipality), Changsha (Hunan Province), Nanchang (Jiangxi Province), Kunming (Yunnan Province), Yulin (Guangxi Zhuang Autonomous Region), and Foshan (Guangdong Province). These sites represent the principal chilli pepper production regions and span a wide range of agro-climatic zones, with diverse climatic conditions, soil types, and cultivation practices, which include both greenhouse and open-field production systems. All agronomic and crop management practices, including pesticide applications and crop protection practices, were standardized in strict accordance with the Guidelines for Pesticide Residue Trials (NY/T 788–2018) as well as the Guidelines for Pesticide Residue Trial Regions issued by the Ministry of Agriculture and Rural Affairs of the People’s Republic of China. Plots used for the terminal residue and dissipation studies were maintained as independent experimental units to prevent cross-interference.

A 30% suspension concentrate (SC) formulation containing fluxapyroxad and trifloxystrobin was through via foliar spraying at the recommended dose of 135 g a.i.ha (grams of active ingredient per hectare), with two applications 7 days apart. Each treated plot was at least 50 m^2^, while a corresponding untreated control plot of the same dimensions received an equal volume of water. A buffer zone of at least 1 m separated the treated and control plots.

Terminal Residue Monitoring. At each of the 12 experimental sites, terminal residue monitoring was conducted on two independent treated plots (each ≥50 m^2^) and one untreated control plot, separated by buffer zones to prevent cross-contamination. Pepper samples were collected at 5 and 7 days after the final application, corresponding to pre-harvest intervals (PHIs) of 5 and 7 days, for terminal residue analysis.

Dissipation Study. To better understand the degradation characteristics of fluxapyroxad and trifloxystrobin under various environmental conditions, dissipation studies were carried out at four representative locations: Taiyuan (Shanxi Province), Qingdao (Shandong Province), Changsha (Hunan Province), and Yulin (Guangxi Zhuang Autonomous Region). Automatic weather stations were installed at each site to continuously record temperature, relative humidity and precipitation throughout the sampling period. Chilli pepper samples were collected 2 h after application, 3, 5, 7, and 10 days after application to characterize dissipation kinetics and calculate the dissipation half-life (DT50).

Sample collection was conducted in accordance with NY/T 788-2018 [20] (Guideline on Pesticide Residue Trials in China). The collection protocol was as follows: at each sampling time point, at least 24 pepper fruits were collected from at least 12 randomly selected locations per plot, covering the upper, middle, and lower parts of the plants; diseased, damaged, or abnormally developed fruits were excluded. Each field sample weighed no less than 2 kg. After collection, pedicels and calyxes were removed, and the whole fruits were cut into approximately 1 cm pieces using a stainless steel knife. A subsample of approximately 200 g was then obtained by quartering, sealed in polyethylene bags, and stored at −20 °C until analysis.

### 2.3. Extraction and Cleanup

An optimized QuEChERS method, modified from the original procedure reported by Anastassiades et al. [21] was used for the extraction and cleanup of fluxapyroxad and trifloxystrobin from chilli pepper samples. The chilli pepper sample (10.00 ± 0.01 g) was weighed into a 50 mL plastic centrifuge tube. Then, 20 mL of acetonitrile containing 5% formic acid (*v*/*v*) was added, along with a ceramic homogenization bead. The mixture was vortexed at 2500 r/min for 10 min. Subsequently, 4 g of anhydrous magnesium sulfate, 1 g of sodium chloride, 0.5 g of disodium hydrogen citrate, and 1 g of sodium citrate were added. The mixture was vortexed at 2500 r/min for 5 min and then centrifuged at 10,600 r/min for 5 min.

A 1.5 mL aliquot of the supernatant was transferred to a 2 mL cleanup tube containing 142.5 mg of anhydrous magnesium sulfate, 20 mg of PSA, and 7.5 mg of GCB. The cleanup tube was vortexed at 2500 r/min for 5 min and centrifuged at 5000 r/min for 2 min. The resulting supernatant was filtered through a 0.22 μm organic membrane filter, transferred to an autosampler vial, and analyzed via UPLC–MS/MS.

### 2.4. Instrument Analysis

Chromatographic separation was performed using a Waters ACQUITY UPLC system equipped with an ACQUITY UPLC HSS T3 column (2.1 × 100 mm, 1.8 μm; Waters, Milford, MA, USA). The column and autosampler temperatures were maintained at 40 °C and 15 °C, respectively. The mobile phase consisted of (A) 4 mmol/L ammonium acetate in water containing 0.1% formic acid and (B) methanol. The gradient elution program was as follows: 0–0.5 min, 90% A; 0.5–3.5 min, linear decrease to 30% A; 3.5–4.8 min, hold at 30% A; 4.8–4.9 min, linear decrease to 5% A; 4.9–6.8 min, hold at 5% A; 6.8–6.9 min, linear increase to 90% A; 6.9–8.5 min, hold at 90% A for re-equilibration. The flow rate and injection volume were 0.3 mL/min and 2 μL, respectively.

Mass spectrometric detection was performed using a Waters Xevo TQ-S micro triple quadrupole mass spectrometer equipped with an electrospray ionization (ESI) source operated in positive ion mode (Waters, Milford, MA, USA). The instrumental parameters were as follows: capillary voltage, 3.0 kV; desolvation temperature, 350 °C; source temperature, 150 °C; cone gas flow, 50 L/h; desolvation gas flow, 800 L/h; collision gas (argon); and multiplier voltage, 650 V. The multiple reaction monitoring (MRM) transitions, cone voltages, and collision energies for each analyte were provided in Table 1.

### 2.5. Method Validation

The analytical method was validated following the “Guideline for Pesticide Residue Testing on Crops” (NY/T 788-2018) [20] and the SANTE/11312/2021 guidelines [22]. The validation parameters assessed included linearity, accuracy, precision, matrix effect, and the limit of quantification (LOQ). Accuracy and precision were evaluated through recovery experiments, in which blank pepper samples were spiked at four concentrations, 0.01, 0.1, 0.5, and 1.0 mg/kg, with five replicates for each level. The limit of quantification (LOQ) was defined, in accordance with SANTE/11312/2021 [22], as the lowest spiking level at which the method achieved acceptable accuracy and precision. For both fluxapyroxad and trifloxystrobin, the LOQ was established at 0.01 mg/kg. Linearity and the matrix effect were assessed by comparing the slopes of matrix-matched calibration curves to those of solvent-based calibration curves. Linearity and matrix effect were evaluated by comparing the slopes of matrix-matched calibration curves with those of solvent-based calibration curves.

### 2.6. Dietary Risk Assessment

Chronic dietary exposure risk was evaluated using the National Estimated Daily Intake (NEDI) model [23], following the methodology recommended by the Joint FAO/WHO Meeting on Pesticide Residues (JMPR) [24]. The NEDI was calculated using Equation (1).

NEDI = Σ(STMRi × Fi) (1)

where STMRi denotes the supervised trial median residue (mg/kg), and Fi represents the dietary consumption of the i-th grade agricultural products for various population groups [24,25]. The average daily consumption data for the general Chinese population were obtained from the 2002 Report on the Nutrition and Health Status of Chinese Residents, a nationally representative survey covering 31 provinces across mainland China [26].

The chronic risk quotient (RQc) was then calculated using Equation (2).

RQc = [NEDI/(ADI × *bw*)] × 100% (2)

where *bw* refers to average body weight, with a default value of 63 kg for adults [26]; ADI denotes the acceptable daily intake (mg/kg bw/day); the ADI values for fluxapyroxad and trifloxystrobin are 0.02 and 0.04 mg/kg bw/day [27], respectively. For residue definitions, fluxapyroxad is defined as the sum of parent fluxapyroxad, M700F008, and M700F048, expressed as fluxapyroxad equivalents. Similarly, trifloxystrobin is defined as the sum of parent trifloxystrobin and CGA 321113, expressed as trifloxystrobin equivalents [28]. An RQc value below 100% indicates negligible or acceptable risk, while a value above 100% signifies an unacceptable risk.

### 2.7. Data Analysis

The dissipation data for fluxapyroxad and trifloxystrobin in chilli peppers were fitted to a first-order kinetic model:

C_t_ = C_0_ e^−kt^ (3)

DT_50_ = ln 2/k (4)

where C_t_ is the residual concentration (mg/kg) at time (t); C_0_ is the initial concentration (mg/kg); (k) is the dissipation rate constant; and DT_50_ is the dissipation half-life (days).

## 3. Results and Discussion

### 3.1. Method Validation

The ubiquity of pesticide residues across environmental and food matrices poses substantial risks to human health, particularly as the toxicity of certain metabolites can equal or even exceed that of their parent compounds [29]. Consequently, the accurate determination of pesticide residues and their transformation products is essential for safeguarding food safety. QuEChERS (quick, easy, cheap, effective, rugged, and safe), originally introduced by Anastassiades et al. [20]. and subsequently standardized as AOAC Official Method,, has become the most widely adopted sample preparation approach for pesticide residue analysis in agricultural products, owing to its simplicity, high throughput, and low solvent consumption. Several analytical strategies have been established for quantifying fluxapyroxad and trifloxystrobin in agri-food products. For instance, Zhu et al. [30] determined trifloxystrobin and kresoxim-methyl in citrus fruits using QuEChERS extraction coupled with HPLC-UV; however, UV detection frequently lacks the sensitivity and selectivity required for trace-level quantification in complex matrices. Alternatively, Zhang et al. [31] and Kang et al. [32] used modified QuEChERS combined with GC-MS/MS to monitor trifloxystrobin and related strobilurin fungicides in fruits, vegetables, and soil. Although trifloxystrobin is amenable to GC separation, fluxapyroxad is preferentially analyzed via LC-MS/MS; thus, a standalone GC-based method is unsuitable for their concurrent determination. In contrast, both fungicides exhibit high ionization efficiency in positive electrospray ionization (ESI+) mode, enabling their simultaneous quantification within a rapid 5 min chromatographic run—an outcome unattainable with HPLC-UV and impractical with GC-based systems. Because chilli pepper represents a challenging matrix rich in pigments and capsaicinoids, rigorous sample cleanup is imperative. In this study, QuEChERS extraction coupled with dispersive solid-phase extraction (d-SPE) using primary–secondary amine (PSA) and graphitized carbon black (GCB) effectively eliminated co-extracted interferences, demonstrating the robustness of the proposed method for the simultaneous determination of both fungicides in this commodity.

#### 3.1.1. Linearity and Matrix Effects

The matrix-matched calibration curves demonstrated strong linearity across the concentration ranges of 0.002–0.07 mg/L for fluxapyroxad and trifloxystrobin, and 0.002–0.2 mg/L for M700F008, M700F048, and CGA 321113. As shown in Table 2, the correlation coefficients (r) for these curves ranged from 0.9940 to 0.9996, indicating that they were adequate for monitoring residues and assessing regulatory compliance for all analytes.

Significant matrix effects were observed for all target compounds in the chilli pepper matrix, ranging from −73.4% for M700F008 to −32.2% for M700F048 (Table 2). This notable suppression aligns with the complex phytochemical composition of pepper fruits, which are rich in pigments, capsaicinoids, and lipophilic co-extractives. These factors can competitively affect ionization efficiency in the electrospray ionization (ESI) source [33,34]. To mitigate these matrix effects and ensure accurate quantification, matrix-matched calibration standards were utilized throughout the entire analytical workflow.

#### 3.1.2. Recovery and Precision

The method’s accuracy and precision were assessed through recovery studies at four fortification levels (0.01, 0.1, 0.5, and 1.0 mg/kg), each with five replicates. As shown in Table 3, mean recoveries for all analytes ranged from 92% to 101%, with RSDs ranging from 2% to 6%. These values were well within the acceptance criteria of NY/T 788-2018 [21] and SANTE/11312/2021 [22], confirming the method’s reliability and reproducibility for simultaneously determining fluxapyroxad, trifloxystrobin, and their metabolites in chilli pepper samples.

### 3.2. Dissipation Kinetics

**Figure 1:**
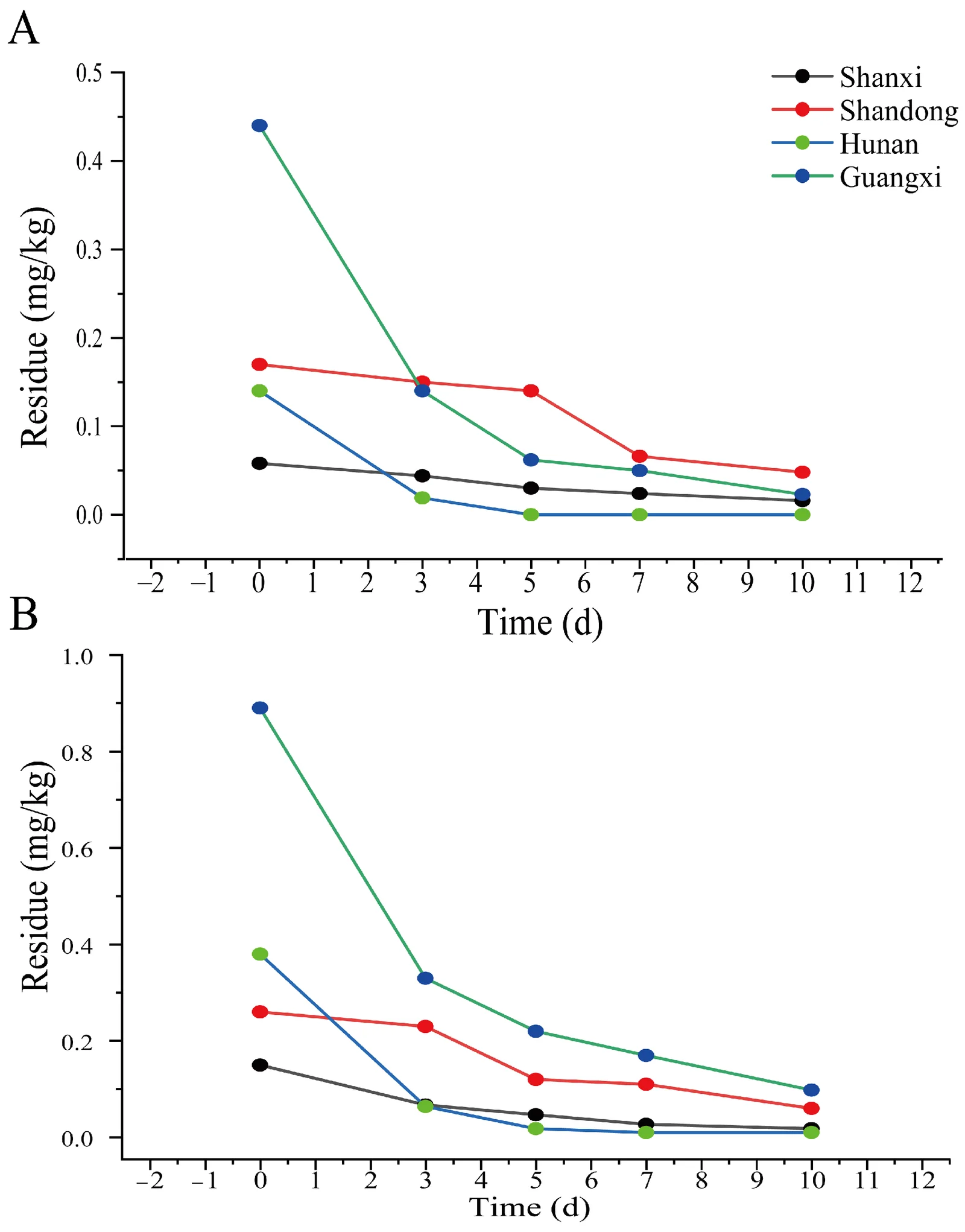
Dissipation kinetics of (**A**) fluxapyroxad and (**B**) trifloxystrobin in chilli peppers under open-field conditions at four representative sites.

After foliar application, residues of both fluxapyroxad and trifloxystrobin declined at all experimental sites, though dissipation rates varied with local microclimatic conditions (Figure 1). Metabolites M700F008, M700F048, and trifloxystrobin acid were absent at the Shanxi, Shandong, and Hunan sites. In Guangxi, both M700F008 and trifloxystrobin residues were detected at day 0 (0.010 and 0.012 mg/kg, respectively), but rapidly degraded to below the LOQ (0.01 mg/kg) by day 10. The highest initial concentrations of fluxapyroxad (0.44 mg/kg) and trifloxystrobin (0.88 mg/kg) were found in Guangxi, while Shanxi showed the lowest (0.058 mg/kg and 0.15 mg/kg, respectively). Both compounds dissipated according to first-order kinetics (Table 4). For the Hunan site, the residue of fluxapyroxad declined below the limit of quantification (LOQ) at 5 days after application; with fewer than five valid data points above LOQ, a degradation kinetic curve could not be reliably fitted. DT_50_ ranged from 2.4 to 5.2 days for fluxapyroxad and from 1.8 to 4.6 days for trifloxystrobin. The dissipation of trifloxystrobin was most rapid in Hunan (DT_50_ = 1.8 days), with residue levels falling below the LOQ within 7 days after application. This rapid loss was mainly attributed to higher temperatures and frequent rainfall, which caused physical wash-off of surface-deposited pesticide via raindrop impact. Collectively, these results indicate that initial deposition efficiency, rather than inherent degradation potential, drove the differing residue profiles among sites.

### 3.3. Terminal Residues of Fluxapyroxad and Trifloxystrobin in Chilli Pepper

**Figure 2:**
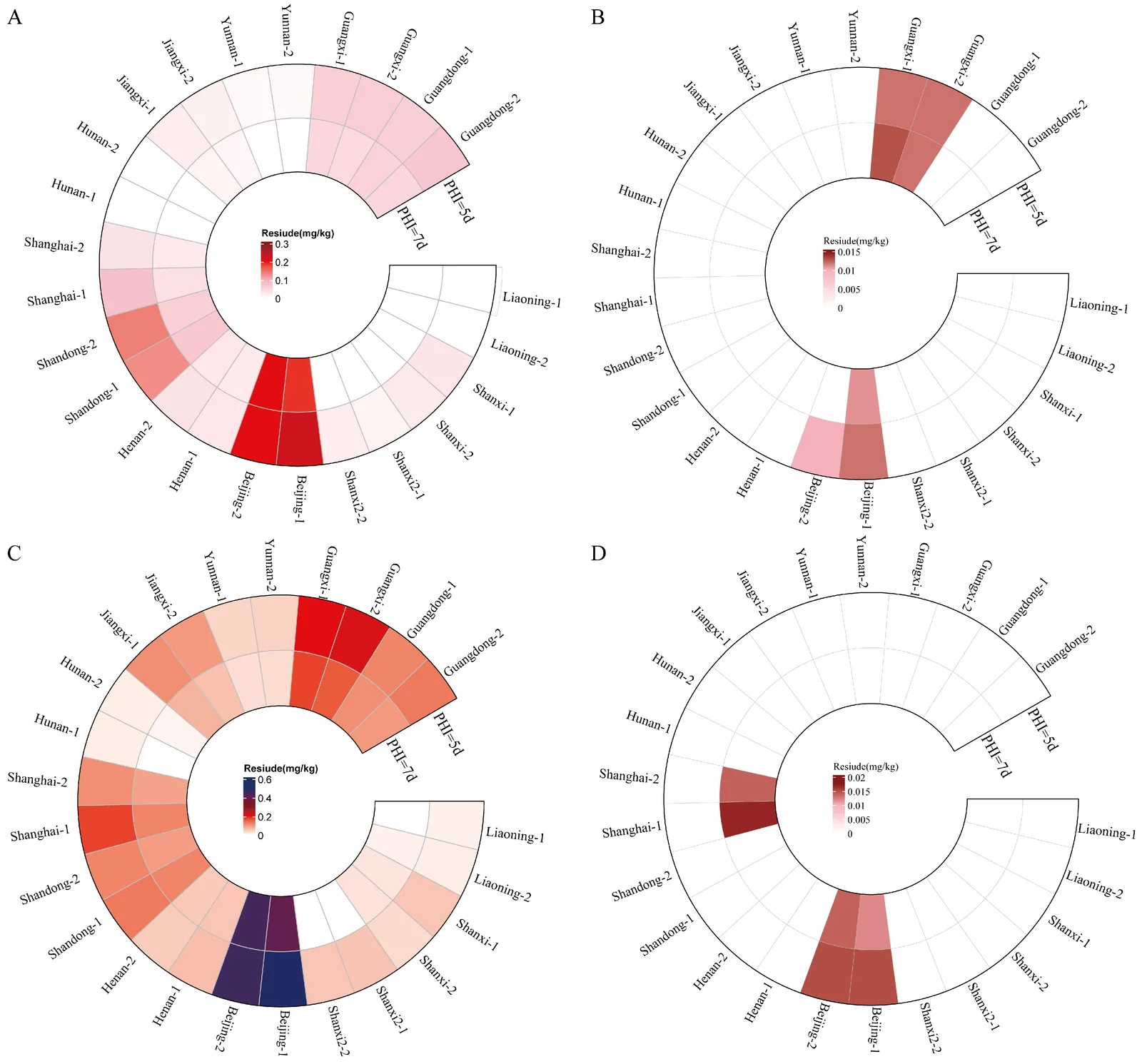
Color map of pesticides detected in pepper samples. (**A**) fluxapyroxad; (**B**) M700F008; (**C**) trifloxystrobin; (**D**) CGA 321113.

Terminal residue levels of fluxapyroxad, trifloxystrobin, and their metabolites in chilli pepper samples collected at 5 and 7 days after the final application across the 12 trial locations were presented in Figure 2. At 5 days post-application, fluxapyroxad residue concentrations ranged from <0.010 to 0.23 mg/kg, with STMR of 0.032 mg/kg and a highest residue (HR) of 0.23 mg/kg. The metabolite M700F008 was detected at trace levels (ranging from <0.010 to 0.012 mg/kg), while M700F048 remained below the LOQ (0.01 mg/kg) at all sites. The total fluxapyroxad residue (the sum of parent and metabolites, as fluxapyroxad equivalents) ranged from 0.010 to 0.24 mg/kg. For trifloxystrobin, the parent compound residues ranged from 0.014 to 0.49 mg/kg (STMR: 0.082 mg/kg; HR: 0.49 mg/kg), whereas its metabolite CGA 321113 was detected at concentrations from <0.010 to 0.015 mg/kg. The total trifloxystrobin residue ranged from 0.014 to 0.51 mg/kg. At 7 days post-application, residue levels generally declined, consistent with natural dissipation processes. Fluxapyroxad residues ranged from <0.010 to 0.21 mg/kg (STMR: 0.026 mg/kg; HR: 0.21 mg/kg), and trifloxystrobin residues ranged from <0.010 to 0.45 mg/kg (STMR: 0.059 mg/kg; HR: 0.45 mg/kg). The metabolites M700F008, M700F048, and CGA 321113 remained at low concentrations, comparable to those recorded at the 5-day sampling point. Notably, the residues of both parent compounds at 7 days were consistently lower than their respective HR values at 5 days, indicating that a 7-day pre-harvest interval (PHI) effectively reduces the residue load before harvest.

Terminal residue levels of fluxapyroxad and trifloxystrobin in chilli peppers varied substantially across the 12 trial regions in China, reflecting the collective influence of regional climate, cropping systems, and agronomic practices. As shown in Figure 2, at a pre-harvest interval (PHI) of 5 days, fluxapyroxad residues ranged from <0.010 mg/kg (Liaoning and Hunan) to 0.23 mg/kg (Beijing), whereas trifloxystrobin residues ranged from 0.014 mg/kg (Liaoning) to 0.49 mg/kg (Beijing); at a 7-day PHI, the corresponding ranges were <0.010–0.21 mg/kg and 0.014–0.45 mg/kg, respectively. Two consistent patterns emerged from these data. First, the highest residues of both fungicides were invariably recorded in Beijing, which may be attributed to several site-specific factors. The trials at this site were conducted under greenhouse conditions, where reduced weathering slowed pesticide dissipation. In addition, a spiral (screw) pepper cultivar with a highly wrinkled surface was grown at this site, and such morphological features are known to trap more pesticide residues than smooth-surfaced cultivars [35]. Moreover, the average temperature during the experimental period was only 1.06 °C, substantially lower than the >10 °C recorded at all other sites, thereby further slowing residue degradation [36,37]. Second, trifloxystrobin residues were consistently higher than those of fluxapyroxad across all regions (approximately 1.7–2.9-fold). This disparity likely results from trifloxystrobin’s higher lipophilicity and extremely low water solubility, which enhance its binding to plant epicuticular waxes and promote strong adhesion to leaf surfaces, thereby reducing losses from leaching and photolysis [38,39,40]. Beyond these site- and compound-specific effects, a distinct north–south geographic gradient was observed. In northern China (Liaoning, Shanxi, Shaanxi, Beijing, Henan, and Shandong), mean residues at 5 and 7 days were 0.075 and 0.055 mg/kg for fluxapyroxad and 0.130 and 0.106 mg/kg for trifloxystrobin, whereas southern China (Shanghai, Hunan, Jiangxi, Yunnan, Guangxi, and Guangdong) exhibited lower mean residues of 0.037 and 0.027 mg/kg for fluxapyroxad and 0.107 and 0.080 mg/kg for trifloxystrobin. These higher residues in the north are likely due to lower ambient temperatures and reduced solar radiation, which attenuate chemical and photolytic degradation. Among individual sites, Shandong, Guangxi, and Guangdong also showed relatively high residues, whereas Liaoning and Hunan remained below the limit of quantification (LOQ, 0.010 mg/kg) at both PHIs. Across all regions, residue concentrations decreased by 15–50% when the PHI was extended from 5 to 7 days. All detected levels were below the corresponding maximum residue limits (MRLs), indicating that the recommended application regime of this fungicide mixture on peppers poses a minimal dietary risk to Chinese consumers.

Maximum residue limits (MRLs) were typically estimated from field trial data according to the Code of Federal Regulations and NY/T 788-2018 guidelines, which recommend setting MRLs at roughly 2 to 4 times the highest residues observed under GAP conditions. According to GB 2763-2026 [41], the MRL for trifloxystrobin is 0.5 mg/kg, nearly equivalent to the residue detected at a 7-day pre-harvest interval (PHI) in this study (0.45 mg/kg). For fluxapyroxad, an MRL of 0.5 mg/kg in chilli pepper was recommended for regulatory consideration. Overall, these results clarify how application methods, weather conditions, and fruit morphology together shape pesticide residue dynamics, providing a solid scientific foundation for dietary risk assessment and regulatory policy.

### 3.4. Chronic Dietary Intake Risk Assessment

**Figure 3:**
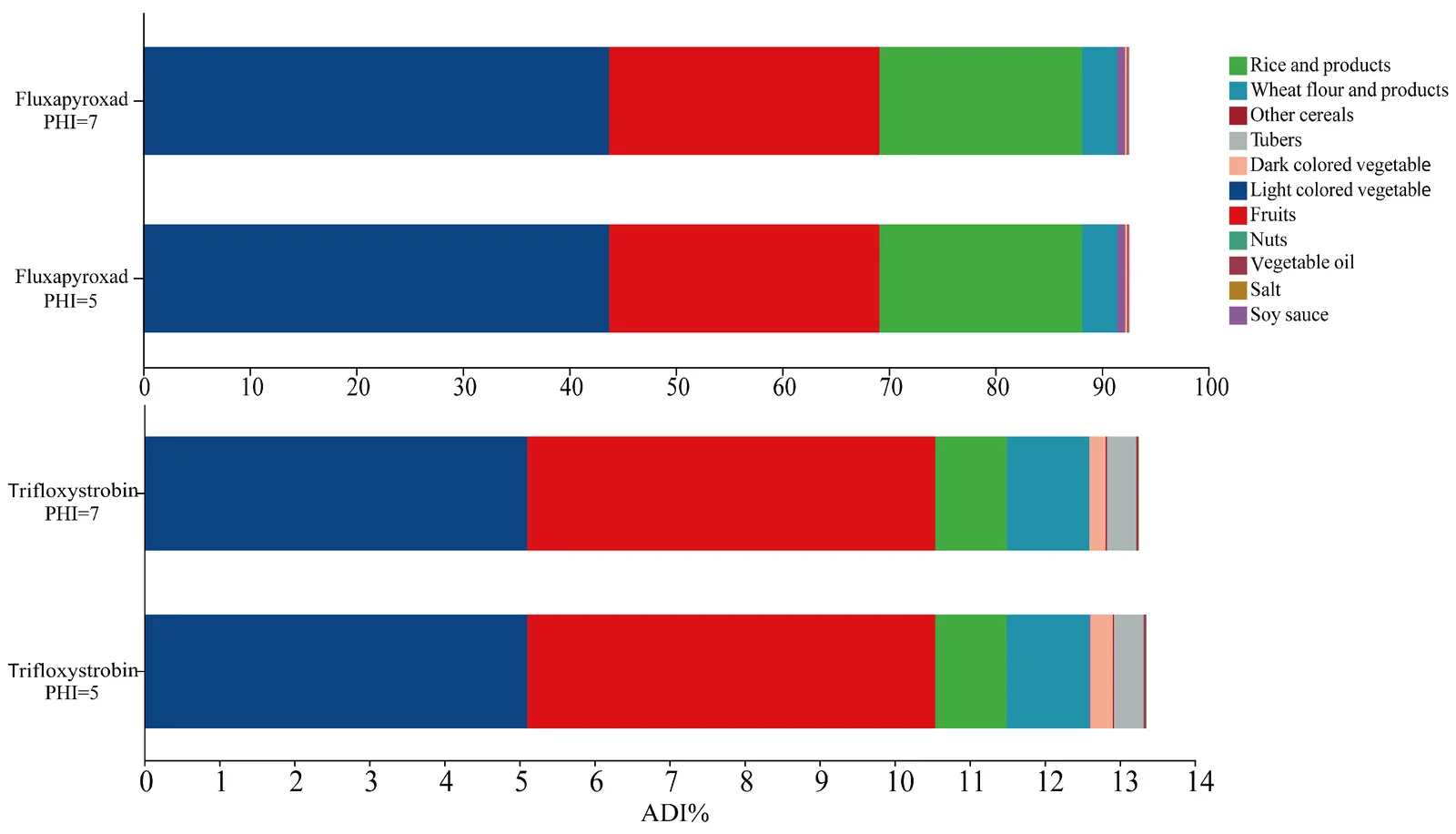
Contribution of different food categories to the dietary risk (ADI%) of fluxapyroxad and trifloxystrobin in pepper at pre-harvest intervals (PHIs) of 5 and 7 days.

The chronic dietary risks of fluxapyroxad and trifloxystrobin were evaluated using STMR values derived from this study together with the food consumption patterns of the general Chinese population (Table 5). Tables S1 and S2 present the MRL or STMR values, daily consumption, and resulting NEDI values for each crop category, while Figure 3 shows the percentage contribution of each crop category to the ADI. In accordance with the residue definitions established by the JMPR [23] and GB 2763-2026 [27], total fluxapyroxad exposure was calculated as the sum of the parent compound and its metabolites (M700F008 and M700F048), expressed as fluxapyroxad equivalents. The main contributing food categories were light-colored vegetables, fruits, and rice and its products. For fluxapyroxad, NEDI values were 1.167 and 1.166 mg, representing 92.61% (5 days) and 92.57% (7 days) of the ADI for a 63 kg adult. The disaggregated results indicate that, for fluxapyroxad, chilli pepper contributed 0.23% (both PHI = 5) and 0.19% (PHI = 7), whereas light-colored vegetables, fruits, and rice and its products were the primary contributors, accounting for 88.17% of the ADI. Previous studies have reported markedly lower ADI risk quotients for fluxapyroxad in other crops, such as apples [42], cucumbers [43], and peanut kernels [44]. These results suggest that chronic dietary exposure to fluxapyroxad from chilli peppers and other registered crops is unlikely to pose a health risk to the Chinese population. For trifloxystrobin, NEDI values were 0.336 and 0.334 mg, accounting for 13.30% (5 days) and 13.27% (7 days) of the ADI. Comparable field studies found even lower risks, with tomatoes at twice the recommended dose yielding risks below 3.38% [45] and brown rice at 11.9% of the ADI [46]. These food categories reflect both higher residue levels and large consumption volumes in the Chinese diet. Importantly, all RQc values remain well below the 100% threshold, indicating a substantial safety margin for chronic dietary exposure.

Taken together, the dietary risk assessment shows that applying 30% fluxapyroxad·trifloxystrobin SC to chilli peppers at the recommended dose (135 g a.i.ha), with a 7-day and a 5-day pre-harvest interval (PHI), does not pose an unacceptable chronic dietary risk to the Chinese population. The RQc for fluxapyroxad was notably higher than for trifloxystrobin, primarily due to fluxapyroxad’s broader crop registrations and the resulting greater dietary exposure. The deterministic approach, consistent with international guidelines, is deliberately conservative and assumes all commodities contain residues at the STMR or the MRL. Likewise, compared with cucumber and brown rice, the higher RQc values in this study mainly reflect higher residue levels in chilli pepper and distinct consumption patterns. Nevertheless, all RQc values remain below the 100% ADI threshold, confirming food safety under the assessed conditions.

## 4. Conclusions

This study assessed the dissipation, residue levels, and chronic dietary exposure risks of fluxapyroxad and trifloxystrobin in peppers following application of a 30% SC co-formulation at 12 field sites across China. The validated QuEChERS-UPLC-MS/MS method enabled precise and simultaneous quantification of both parent compounds and their metabolites (M700F008, M700F048, and CGA 321113), meeting all regulatory standards for linearity, sensitivity, accuracy, and precision. Both fungicides showed rapid dissipation in peppers, following first-order kinetics with DT50 of 2.4–5.2 days for fluxapyroxad and 1.8–4.6 days for trifloxystrobin. At the recommended dose, terminal residue trials found fluxapyroxad residues of <0.010 to 0.23 mg/kg and trifloxystrobin residues of <0.010 to 0.49 mg/kg. Dietary risk assessment using the NEDI model showed risk quotients of 92.57–92.61% of the ADI for fluxapyroxad and 13.27–13.30% for trifloxystrobin, both well below the 100% safety threshold. These findings confirm that, under the tested GAP conditions, the co-formulation poses no unacceptable chronic dietary risk to the general Chinese population. To our knowledge, this is the first systematic, multi-regional investigation of the dissipation and residues of this co-formulation in chilli peppers in China. Future studies should determine processing factors for dried and pickled chilli products, extend the method to other matrices and related SDHI/QoI fungicides and evaluate cumulative dietary exposure to this fungicide combination, and verify the method’s robustness through inter-laboratory validation.

## Figures and Tables

**Table 1:** The MRM parameters for the target compounds.

Compound	Chemical Structures	Retention Time (min)	Precursor Ion (*m*/*z*)	Product Ion (*m*/*z*)	Cone Voltage (V)	Collision Energy (V)
Fluxapyroxad	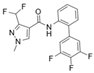	6.22	382.1	362.0 ^a^	30	18
				342.0		20
M700F008	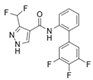	6.07	368.2	348.1 ^a^	93.5	23.3
				328.1		34.4
M700F048	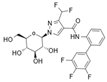	5.45	530.1	368.1 ^a^	82.3	21.8
				348.0		39.0
Trifloxystrobin	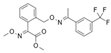	6.58	409.1	186.1 ^a^	40	23
				145.0		63
CGA 321113	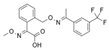	6.41	395.0	186.0 ^a^	80	10
				148.0		10

^a^ Quantitative ion transition.

**Table 2:** Regression parameters and matrix effects for fluxapyroxad, trifloxystrobin, and their metabolites in chilli peppers.

Compound	Matrix	Linear Regression Equation	Coefficient of Determination (R^2^)	Matrix Effects (ME, %)
Fluxapyroxad	Acetonitrile	y = 2.86 × 10^8^x + 4811.7	0.9972	-
	Chilli pepper	y = 9.20 × 10^7^x − 15,629.5	0.9991	−67.8
M700F008	Acetonitrile	y = 1.01 × 10^8^x + 855.1	0.9986	-
	Chilli pepper	y = 2.69 × 10^7^x − 17,409.1	0.9974	−73.4
M700F048	Acetonitrile	y = 2.76 × 10^7^x − 8218.8	0.9989	-
	Chilli pepper	y = 1.87 × 10^7^x − 2689.9	0.9996	−32.2
Trifloxystrobin	Acetonitrile	y = 3.32 × 10^8^x + 41,700.1	0.9940	-
	Chilli pepper	y = 1.94 × 10^8^x + 77,561.1	0.9977	−41.6
Trifloxystrobin acid	Acetonitrile	y = 5.78 × 10^6^x + 2907.2	0.9946	-
	Chilli pepper	y = 3.17 × 10^6^x − 836.1	0.9995	−45.2

**Table 3:** Recoveries and relative standard deviations (RSDs) of fluxapyroxad, trifloxystrobin, and their metabolites in chilli pepper at four fortification levels (n = 5).

Compound	0.01 mg/kg		0.1 mg/kg		0.5 mg/kg		1 mg/kg	
	Recoveries (%)	RSD (%)	Recoveries (%)	RSD (%)	Recoveries (%)	RSD (%)	Recoveries (%)	RSD (%)
Fluxapyroxad	98	2	98	2	100	5	98	3
M700F008	97	2	96	2	95	5	94	4
M700F048	93	2	94	2	96	4	92	4
Trifloxystrobin	92	4	100	4	99	6	98	3
Trifloxystrobin acid	99	5	101	3	100	5	93	5

**Table 4:** Dissipation kinetic equations and half-lives of fluxapyroxad and trifloxystrobin in chilli pepper at four representative sites.

Site	Fluxapyroxad			Trifloxystrobin		
	Equation (C_t_=)	Coefficient of Determination (R^2^)	DT_50_ (d)	Equation (C_t_=)	Coefficient of Determination (R^2^)	DT_50_ (d)
Shanxi	0.0603e^−0.132t^	0.9906	5.2	0.1371e^−0.214t^	0.9846	3.2
Shandong	0.2037e^−0.137t^	0.8629	5.1	0.2916e^−0.152t^	0.9376	4.6
Hunan	/	/	/	0.2229e^−0.378t^	0.8591	1.8
Guangxi	0.3615e^−0.290t^	0.967	2.4	0.7345e^−0.212t^	0.9658	3.3

**Table 5:** Chronic dietary risk assessment of fluxapyroxad and trifloxystrobin based on total residue (parent + metabolites) for the Chinese general population.

Compound	ADI (mg/kg bw/day)	5 Days After Final Application			7 Days After Final Application		
		STMR (mg/kg)	NEDI (mg)	RQc (%)	STMR (mg/kg)	NEDI (mg)	RQc (%)
Fluxapyroxad	0.02	0.032	1.167	92.61	0.026	1.166	92.57
Trifloxystrobin	0.04	0.082	0.336	13.30	0.059	0.334	13.27

## Data Availability

The original contributions presented in the study are included in the article/Supplementary Materials; further inquiries can be directed to the corresponding author.

## Supplementary Materials

The following supporting information can be downloaded at: https://www.mdpi.com/article/10.3390/foods15213818/s1, Table S1: National Estimated Daily Intake (NEDI) of fluxapyroxad for the Chinese general population; Table S2: National Estimated Daily Intake (NEDI) of trifloxystrobin for the Chinese general population; Table S3: The information on field trial; Table S4: Terminal residues of fluxapyroxad, trifloxystrobin, and their metabolites in pepper across 12 trial locations (mg/kg).
