# Supplementary material for: Dissipation, Residues and Dietary Risk Assessment of Fluxapyroxad and Trifloxystrobin in Chilli Peppers
Source: Foods. 2026 Oct 26;15(21):3818. doi: 10.3390/foods15213818 (PMC13762926; doi:10.3390/foods15213818)
Supplement: Supplementary file 1 [file foods-15-03818-s001.zip › foods-4610380-supplementary.pdf]

Table S1. National Estimated Daily Intake (NEDI) of fluxapyroxad for the Chinese general population

Food Category	Consumption (kg/day)	Reference Limit (mg/kg)	Limit Source	NEDI (mg)
Rice and products	0.2399	1.0	China	0.2399
Wheat flour and products	0.1385	0.3	China	0.0416
Other cereals	0.0233	0.05	China	0.0012
Tubers	0.0495	0.02	China	0.0010
Dark-colored vegetables	0.0915	0.032(PHI=5)<br>0.026(PHI=7)	STMR(this study)	0.0029(PHI=5)<br>0.0024(PHI=7)
Light-colored vegetables	0.1837	3.0	China	0.5511
Fruits	0.0457	7.0	CAC(berries)	0.3199
Vegetable oil	0.0327	0.01	China	0.0003
Soy sauce	0.0090	1.0	Korea	0.0090

Table S2. National Estimated Daily Intake (NEDI) of trifloxystrobin for the Chinese general population

Food Category	Consumption (kg/day)	Reference Limit (mg/kg)	Limit Source	NEDI (mg)
Rice and products	0.2399	0.1	China	0.0240
Wheat flour and products	0.1385	0.2	China	0.02777
Other cereals	0.0233	0.02	China	0.0005
Tubers	0.0495	0.2	China	0.0099
Dark-colored vegetables	0.0915	0.082(PHI=5)<br>0.059(PHI=7)	STMR(this study)	0.0075(PHI=5)<br>0.0054(PHI=7)
Light-colored vegetables	0.1837	0.7	China	0.1286
Fruits	0.0457	3.0	CAC(berries)	0.1371
Nuts	0.0039	0.02	China	0.0001
Vegetable oil	0.0327	0.02	China	0.0007
Salt	0.0120	0.01	CAC	0.0001
Soy sauce	0.0090	0.01	CAC	0.0003

Table S3. The information on field trial

Location(Province)	Cultivation	Soil properties			Climate	
		pH	OMC (%)	CEC (cmol/kg)	Average temperature (℃)	Rainfall （mm）
Shenyang (Liaoning province)	Field	7.70	0.8%	19.5	25.10	307.7
Taiyuan (Shanxi province)	Greenhouse	8.71	3.93%	12.55	27.35	24.4
Weinan (Shanxi province)	Greenhouse	7.88	0.95%	4.70	14.80	57.3
Changping (Beijing)	Greenhouse	7.32	5.38%	23.70	1.06	3.3
Xinxiang (Henan provincce)	Greenhouse	8.76	0.965%	8.61	27.67	4.0
Qingdao (Shandong province)	Greenhouse	5.78	1.15%	17.8	28.33	168.2
Songjiang (Shanghai)	Greenhouse	6.70	4.28%	14.39	14.65	7.2
Changsha (Hunan province)	Field	5.10	2.80%	6.70	31.08	-

Nanchang (Jiangxi province)	Field	5.75	2.03%	3.70	13.03	18.8
Kunming (Yunnan province)	Field	-	-	-	22.13	921.23
Yulin (Guangxi province)	Field	6.62	1.95%	4.60	23.83	19.2
Foshan (Guangdong province)	Field	5.90	1.02%	3.90	18.63	14.3

OMC: organic matter content; CEC: cation exchange capacity.

Table S4. Terminal residues of fluxapyroxad, trifloxystrobin, and their metabolites in pepper across 12 trial locations (mg/kg)

Compound	5 Days After Final Application			7 Days After Final Application		
	Range (mg/kg)	STMR (mg/kg)	HR (mg/kg)	Range (mg/kg)	STMR (mg/kg)	HR (mg/kg)
Fluxapyroxad	<0.010–0.23	0.032	0.23	<0.010–0.21	0.026	0.21
M700F008	<0.010–0.012	0.010	0.012	<0.010–0.013	0.010	0.013
M700F048	<0.010	0.010	0.010	<0.010	0.010	0.010
Total Fluxapyroxad[a]	0.010–0.24	0.032	0.24	<0.010–0.21	0.026	0.21
Trifloxystrobin	0.014–0.49	0.082	0.49	0.014–0.45	0.059	0.45
Trifloxystrobin acid	<0.010–0.015	0.010	0.015	<0.010–0.017	0.010	0.017
Total Trifloxystrobin[b]	0.014–0.51	0.082	0.51	<0.010–0.46	0.059	0.46
